# Pleuropulmonary Blastoma in Children: A Nationwide Multicenter Study

**DOI:** 10.3390/cancers17193223

**Published:** 2025-10-02

**Authors:** Barbara Tejza, Marta Hetman, Jadwiga Węcławek-Tompol, Krzysztof Kałwak, Olga Rutynowska, Bożenna Dembowska-Bagińska, Agata Sobocińska-Mirska, Paweł Łaguna, Ewa Bień, Ninela Irga-Jaworska, Katarzyna Derwich, Agnieszka Wziątek, Katarzyna Pawińska-Wąsikowska, Walentyna Balwierz, Anna Pytlik, Katarzyna Drabko, Justyna Walenciak, Wojciech Młynarski, Marta Rzeszutko, Jan Styczyński

**Affiliations:** 1 Department of Pediatric, Hematology and Oncology, Jurasz University Hospital, Collegium Medicum Bydgoszcz, Nicolaus Copernicus University Toruń, 85-094 Bydgoszcz, Poland; jstyczynski@cm.umk.pl; 2 Department of Pediatric Bone Marrow Transplantation, Oncology and Hematology, Wroclaw Medical University, 50-556 Wroclaw, Poland; martha.hetman@usk.wroc.pl (M.H.); jadwiga.weclawek-tompol@usk.wroc.pl (J.W.-T.); krzysztof.kalwak@umw.edu.pl (K.K.); 3 Department of Oncology, The Children’s Memorial Health Institute, 04-730 Warsaw, Poland; o.rutynowska@ipczd.pl (O.R.); b.dembowska@ipczd.pl (B.D.-B.); 4 Department of Oncology, Children’s Hematology, Clinical Transplantology and Pediatrics, University Clinical Center of the Medical University, 02-091 Warsaw, Poland; agata.mirska@uckwum.pl (A.S.-M.); pawel.laguna@wum.edu.pl (P.Ł.); 5 Department of Pediatrics, Hematology and Oncology, Medical University of Gdańsk, 80-211 Gdańsk, Poland; ewa.bien@gumed.edu.pl (E.B.); ninela.irga-jaworska@gumed.edu.pl (N.I.-J.); 6 Department of Pediatric Oncology, Hematology and Transplantology, Jonscher Clinical Hospital, Poznan University of Medical Sciences, 60-572 Poznań, Poland; kderwich@ump.edu.pl (K.D.); awziatek@ump.edu.pl (A.W.); 7 Department of Pediatric, Oncology and Hematology, Jagiellonian University Medical College, 30-663 Cracow, Poland; katarzyna.pawinska-wasikowska@uj.edu.pl (K.P.-W.); balwierz@mp.pl (W.B.); 8 Department of Pediatric Hematology, Oncology and Transplantology, Medical University of Lublin, 20-093 Lublin, Poland; anna.pytlik@uszd.lublin.pl (A.P.); katarzynadrabko@umlub.pl (K.D.); 9 Department of Pediatrics, Oncology, Hematology, Medical University, 91-738 Łódź, Poland; justyna.walenciak@umed.lodz.pl (J.W.); wojciech.mlynarski@umed.lodz.pl (W.M.); 10 Department of of Pathomorphology, Wroclaw Medical University, 50-367 Wroclaw, Poland; rzemarta@wp.pl

**Keywords:** pleuropulmonary blastoma, children, DICER1 syndrome

## Abstract

**Background/Objectives**: This study involved an analysis of clinical data, histological types, genetic predisposition, treatment and outcomes in PPB in children. **Patients and methods**: We conducted a retrospective review of children treated for PPB at Polish pediatric oncology centers between 2011 and 2024. **Results:** A total of fifteen children (seven boys, eight girls; median age of 39 months; range: 27–64 months) were included. Type II solid/cystic PPB and type III solid PPB were diagnosed in six and eight children, respectively (one not known). Overall, 93% of patients were diagnosed at up to 4 years of age. Metastatic disease at diagnosis was confirmed in three (20%) patients, localized in bones, bone marrow and lymph nodes. Diagnosis was confirmed via central pathology review in 11 patients (73%). DICER1 pathogenic variants were identified in eight patients. All children presented with respiratory symptoms. The tumor dimensions were >10 cm (n = 7), 5–10 cm (n = 5) and <5 cm (n = 2). No bilateral lung involvement was observed. Tumor biopsy was performed in six children (40%), with subsequent resection (R0) in five patients. Primary resection (R0) was achieved in three patients (20%) with type II (n = 1) or type III (n = 2). In the other six patients, non-radical resection was performed: R1 in four (27%) children (with a tumor rupture in one patient) and R2 (subtotal resection) in two children (13%). All patients received postoperative chemotherapy. Maintenance chemotherapy was given to two patients. No patient received radiotherapy as first-line treatment. Progressive disease occurred in two patients in the CNS and lungs. Relapsed disease appeared in three patients, all with CNS involvement. **Conclusions**: PPB is a rare, malignant tumor of early childhood with an uncertain prognosis. Despite multimodal treatment, patients remain at risk of progression or CNS relapse. Complete surgical resection remains a key prognostic factor.

## Simple Summary

The aim of the study was to analyze the treatment of pleuropulmonary blastoma (PPB) in a group of 15 pediatric patients, with a median age of 39 months. PPB is a primary, malignant tumor of the lung and pleura in populations of young children. Key elements of treatment include surgical resection and postoperative chemotherapy. Regional and distant metastases have been reported in 20% of patients at diagnosis. Progressive and relapsed disease was observed, all with CNS involvement with a very poor outcome. The clinical manifestation of PPB is nonspecific, and the tumor can be misdiagnosed with congenital lung malformation. Genetic testing towards DICER1 pathogenic variants is recommended to recognize PPB and other DICER1-associated disorders.

## 1. Introduction

Primary lung tumors are rare in pediatric populations. Published reports in the literature indicate limited knowledge on these tumors [1,2,3,4,5]. The estimated incidence of primary lung tumors in pediatrics is 0.049 newly diagnosed cases per 100,000 children per year [6]. Pleuropulmonary blastoma (PPB) is reported to be the most frequent primary lung tumor described in children between 0 and 6 years. The meedian age at diagnosis of PPB is 8.4 months for type I and 41 months for type II/III PPB [7,8,9,10]. Other primary lung tumors in children are very rare and include mucoepidermoid carcinomas, carcinoid tumors and inflammatory and myofibroblastic tumors (IMTs) [11,12,13,14].

PPB is a primary malignant tumor of the lung and pleura occurring in children. It is a rare disease representing 0.2–0.3% of all pediatric cancers, mainly in children up to 7 years of age. PPB belongs to the group of embryonal tumors, similarly to hepatoblastoma, neuroblastoma or nephroblastoma. The earliest reported case of PPB was detected prenatally via ultrasound before 31 weeks of gestation.

The clinical presentation of PPB is often nonspecific, frequently mimicking lower respiratory tract infections such as pleuropneumonia, which do not improve with symptomatic or anti-inflammatory treatment. Pneumothorax is observed in cystic tumors. In case of delayed diagnosis of advanced disease, respiratory insufficiency may occur. A chest X-ray typically reveals an opaque mass accompanied by atelectasis, inflammatory changes in adjacent lung lobes, pleural effusion and contralateral mediastinal shift. The most common initial diagnoses are pleuropneumonia, pleural empyema and lung abscess. Chest computed tomography (CT) allows differentiation of a cystic, solid-cystic or solid tumor mass. In some cases, the radiological image may suggest congenital lung defects of a cystic nature, and the diagnosis of cancer is made after a planned surgical procedure [15,16].

PPB is classified into four histological types (IR, I, II, III), with type I (cystic) associated with the best prognosis and type III (solid) linked to the worst outcomes. Type I PPB occurs as a single or multilocular cyst characterized by a subepithelial layer of primitive malignant mesenchymal cells. The possibility of histological evolution from a cystic lesion to a stage of higher malignancy and the possibility of regression (type IR) are described. It is currently believed that the transition from type I to types II or III requires the coexistence of DICER1 pathogenic variants alongside additional genetic alterations, particularly in the *TP53* and *NRAS* genes. The absence of additional mutations may stop progression, leading to the development of type IR PPB, a purely cystic lesion without malignant cells. In an unfavorable course, the proliferation of neoplastic cells leads to the formation of a solid mass with residual cyst foci (type II) or without (type III). Biopsy material may be insufficient for a proven diagnosis of PPB and its type due to the small amount of tumor tissue. A report from the International PPB/DICER1 Registry was published in 2023. The data and outcomes in the largest-ever reported cohort of 314 children with PPB from 47 countries from 1987 to 2021 were reported. The 5-year overall survival (OS) rates vary, with type IR showing the most favorable prognosis, at 100%, followed by type I, at 98%. For type II PPB, 5-year OS was reported at 79,1% for local and at 40% for distant metastatic disease; for type III, the OS rate was 60% for local disease. All assessable patients with type III and distant metastases at diagnosis died of disease by 2 years. The 5-year PPB-EFS was 96,4% for type IR and 90,3% for type I. EFS for type II was 65,9% for local and 20% for metastatic disease. Five-year EFS for type III and local disease was 46,9%. All assessable patients with type III with distant metastases had a PPB event by 15 months [17,18].

Approximately 60–80% of patients with PPB are carriers of DICER1 pathogenic variants. DICER1 syndrome, familial tumor predisposition disorder, is a genetic predisposition to the development of certain malignant tumors and benign disorders. The DICER1 gene, identified in 2009, encodes the DICER1 protein, playing a key role in the post-transcriptional regulation of mRNA and function of oncogenic proteins. Around 87% are germline mutations with autosomal dominant inheritance and incomplete penetrance of the gene, while about 13% mutations are de novo. Patients with a germline DICER1 pathogenic variants have an increased risk of developing PPB, which is the most common cancer associated with DICER1 syndrome, as well as thyroid disease (multinodular goiter, thyroid cancer), ovarian tumors (Sertoli–Leydig cell tumors), kidney tumors (cystic nephroma, Wilms tumor, anaplastic renal sarcoma) and embryonal rhabdomyosarcoma. Additionally, brain tumors (pineoblastoma, nasal chondromesenchymal hamartoma) may develop, with the highest risk occurring in early childhood and decreasing in adulthood. Patients with a germline DICER1 mutation experience various non-neoplastic disorders, such as macrocephaly, renal defects, retinal abnormalities and dental development disorders. Clinical manifestations of DICER1 mutations, in the form of malignant changes and non-neoplastic disorders, can occur as different variants in one patient or individually. In children with this mutation, close clinical observation is needed for atypical manifestations and imaging studies for early detection of a second tumor. In 2018, the International Pleuropulmonary Blastoma/DICER1 Registry published guidelines for follow-up testing for patients with a proven or suspected germline mutation [19,20,21,22,23,24,25]. Apart from the histological type, the complete removal of the tumor (R0 resection) is the most important prognostic factor. If surgery is not possible, a biopsy is performed to confirm the diagnosis. After chemotherapy and improvement in the surgical conditions, a radical resection of the tumor is attempted again. Complete resection is particularly crucial in type I PPB, as approximately 10% of cases progress to type II or III, markedly worsening the prognosis. The tumor causes local recurrence in the chest and distant metastases, mainly to the brain, spinal cord and bones, both during and after treatment.

The aim of this study was to analyze the clinical characteristics, histological subtypes, genetic background and treatment outcomes in pediatric patients with pleuropulmonary blastoma.

## 2. Materials and Methods

*Study design*. All patients with pleuropulmonary blastoma diagnosed between 2011 and 2024 were analyzed in a retrospective, registry-based study.

*Data collection.* The HOPE database of the Polish Society of Pediatric Oncology and Hematology (PSPOH), which includes patients with soft tissue sarcomas, was the source of core tumor data, including clinical type, diagnostics, therapeutic strategies and patient outcomes. Data were analyzed centrally and verified with the participating centers whenever necessary. No exclusion criteria were applied. The diagnosis of PPB was made locally and confirmed histopathologically and molecularly at a reference center.

*Treatment.* Patients were considered for combination treatment with chemotherapy, surgery or radiotherapy. The chemotherapy regimens were based on treatment recommendations, according to CWS Guidance (i.e., Cooperative Soft Tissue Sarcoma Study Group) in cooperation with the International Pleuropulmonary Blastoma Registry, and on EXPeRT/PARTNER therapeutic recommendations for type II/III PPB (European Cooperative Study Group for Pediatric Rare Tumors within the European Union-funded project Pediatric Rare Tumors Network—European Registry), according to the policy of the PSPOH [26,27].

*Dataset availability.* This study is based on registry data from patients with a confirmed PPB diagnoses. No personal data were included. Given the retrospective, registry-based nature of this study, approval from an ethical review board was not required. The dataset remains the property of PSPOH and may be made available upon reasonable request to the corresponding author.

*Statistical methods.* The primary endpoint was the probability of event-free survival (pEFS), while the probability of overall survival (pOS) was the secondary endpoint. Event was defined as relapse or death. Comparisons of categorical variables were performed using Fisher’s exact test or the χ^2^ test. Survival curves were estimated with the Kaplan–Meier method and compared using the log-rank test. Median follow-up was calculated according to the inverted Kaplan–Meier technique. The univariate and multivariate effects of variables on EFS were analyzed using a Cox proportional hazards model to estimate hazard ratios (HRs) with 95% confidence intervals (95% CIs). All tests were two-sided, with the type I error rate fixed at 0.05. The following factors were included in the risk factor analysis: sex, tumor laterality (side), tumor size, TNM tumor classification (T), TNM lymph node involvement (N), TNM metastasis status (M), extent of initial surgery (R), TNM stage and overall clinical stage, PPB histological type and second surgery status. All the analyses were performed using the statistical software SPSS (IBM, Armonk, NY, USA), v29.

## 3. Results

### 3.1. Demographics

A total of 15 patients diagnosed with PPB between 2011 and 2024 across nine oncology centers were analyzed (Table 1). The study group consisted of fifteen children, including seven boys and eight girls (sex ratio: 1:1.1), aged 27 to 62 months (approximately 2.3 to 5.2 years) at the time of diagnosis. The median age at diagnosis was 39 months (3 years).

### 3.2. Diagnosis

**Figure 1:**
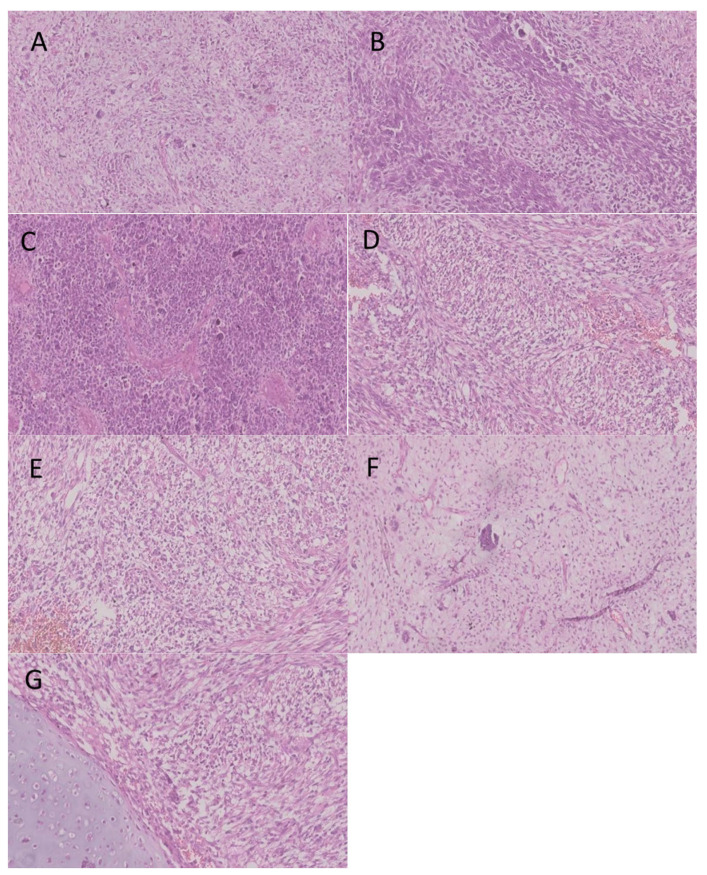
(**A**–**G**) Histopathology images of pleuropulmonary blastoma with a complete overview of the hematoxylin–eosin (HE) staining. The tumor is highly cellular (**A**) (magnification ×10), with solid foci of anaplastic cells and scattered pleomorphic (**B**) (×10) large cells (**C**) (×15) and also few hypocellular areas with myxoid stroma. The areas of necrosis and hemorrhage are large. Spindle sarcomatous cell proliferations are arranged in fascicular patterns (**D**) (×20) with areas of blastematous, rhabdoid (**E**) (×15), liposarcomatous (**F**) (×20) and myxoid components. Tumor cells were non-cohesive with disorganized growth patterns, and little elements of cartilage can be seen (**G**) (×20).

The most common histological subtype was type III PPB (n = 8; 53.3%), with a median age at diagnosis of 39.5 months (over 3 years of age), followed by type II PPB (n = 6; 40%), with a median age of 34 months (under 3 years of age). In one case, the histological type was unknown. Typical histological findings are shown in Figure 1A–G. In 93.3% of cases, the disease was diagnosed before the age of 4 years. The metastatic form was diagnosed in three patients (20%) with localization to the bones, bone marrow and lymph nodes. Diagnosis was centrally confirmed in eleven patients (73%), while in the other four patients, the diagnosis was confirmed locally by two pathologists. DICER1 pathogenic variants were confirmed in eight patients. This was not tested in six patients, and one tested negative.

### 3.3. Symptoms and Signs

All patients presented with respiratory symptoms, including cough, fever and dyspnea. Two children developed respiratory failure and required treatment in an intensive care unit (ICU). In one patient with a congenital lung defect, the diagnosis was made after surgery and histopathological examination. Pneumothorax occurred in two children. Neurological symptoms were present in two children with progressive and relapsed diseases of the CNS. These included headaches, vomiting, balance disorders and limb tremors. In two patients, the lesions were detected during routine follow-up MRI imaging, without accompanying clinical symptoms.

### 3.4. Tumor Localization and Size

**Figure 2:**
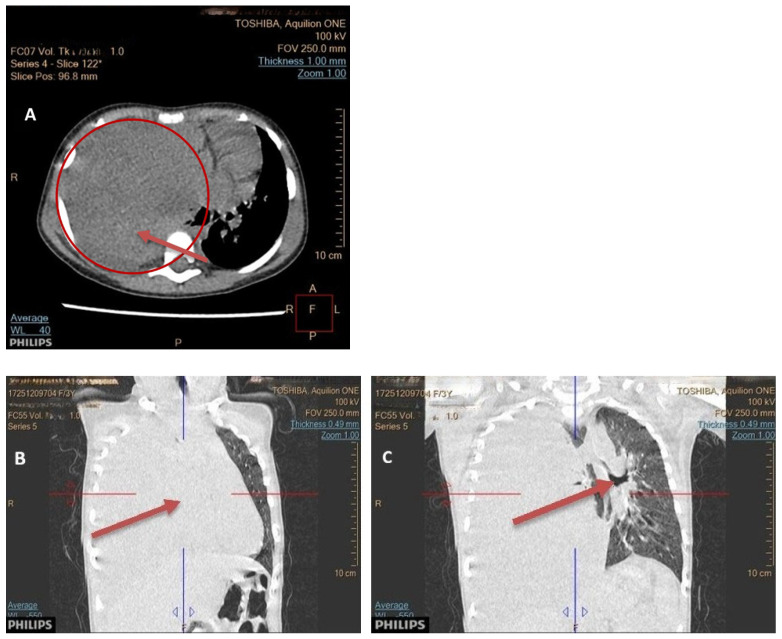
(**A**–**C**) A computed tomography imaging revealing a large, solid mass, at 11 × 9 × 11 cm, in the right hemithorax (**A**). The right lung is compressed and completely atelectatic (**B**). Marked tracheal deviation and mediastinal shift to the left side is noted (**C**).

**Figure 3:**
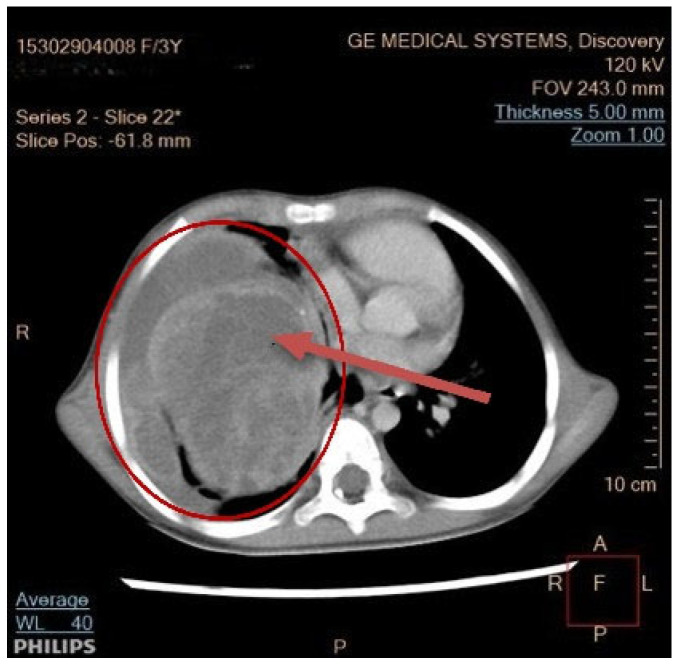
Enhanced contrast of CT scan. The arrow indicates a large 8 × 9 × 16 cm heterogeneously enhancing solid mass in the right hemithorax with pleural effusion.

**Figure 4:**
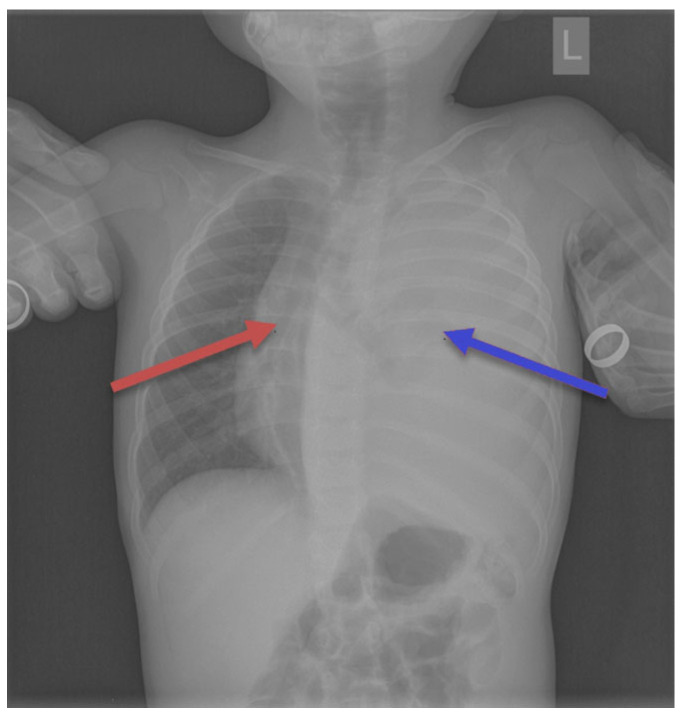
Chest radiograph showed complete homogenous opacification of the left hemithorax (the blue arrow) with contralateral mediastinal and tracheal deviation (the red arrow).

Seven children had a tumor located on the left side of the chest, and eight children on the right side. In seven children, the dimensions of the tumor were >10 cm; in five, they measured 5–10 cm; and in two patients, they were below 5 cm. In one patient, the dimensions were difficult to assess due to the occurrence of a massive pneumothorax. No bilateral lesions were found. Typical radiological findings are shown on Figure 2, Figure 3 and Figure 4.

### 3.5. Treatment

In six cases (40%), the primary surgical procedure was limited to a tumor biopsy. All patients received neoadjuvant chemotherapy, followed by a delayed surgery. According to CWS therapy protocol, surgery was performed after five cycles (four patients) or three cycles (one patient) of VAIA III and after five cycles of I2VAd (one patient). In five patients, postoperative R0 status and complete response (CR) were achieved. One patient did not achieve remission and experienced disease progression within approximately 3 months after diagnosis. Primary radical resection (R0) was performed in three patients (20%), including one with type II and two with type III PPB. The remaining patients underwent a non-radical procedure, while four patients (27%) underwent R1 resection, including one case in which tumor rupture occurred during the procedure. Two patients (13%) underwent a subtotal R2 resection (Table 2). One patient had left-sided pneumonectomy due to pleural, chest wall and hilar invasion. In five patients, a single lobe was removed via thoracotomy or thoracoscopy. All patients received postoperative chemotherapy. One patient received upfront salvage therapy with a COP cycle (cyclophosphamide, vincristine, prednisone) prior to histological confirmation. Two patients received oral maintenance chemotherapy (OTI-OTE); one patient for 5 months with CR after treatment and the other for 3 months. However, they relapsed 1 month after the end of treatment. None of the patients received radiotherapy as a part of first-line treatment. Radiotherapy was administered in two patients at relapse in the central nervous system.

### 3.6. Event-Free Survival

**Figure 5:**
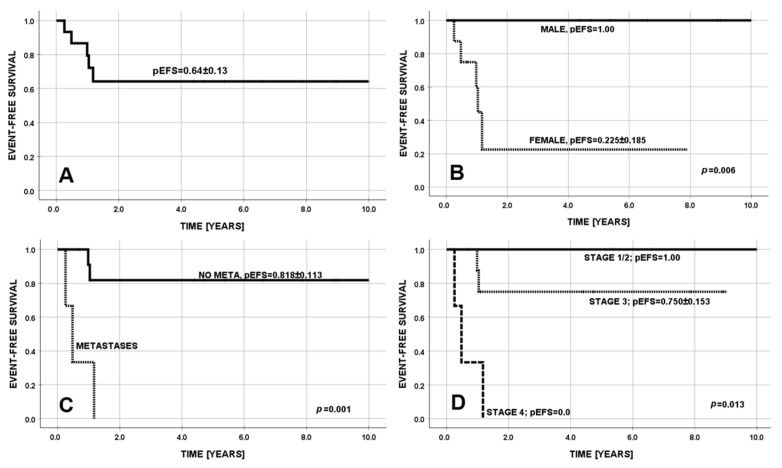
Probability of event-free survival (pEFS): (**A**) all patients (5/15 events) at 2 years (0.64 ± 0.13); (**B**) males vs. females; (**C**) presence of metastases; and (**D**) TNM stage of the disease.

The estimated probability of EFS at 5 years was 0.64 (95%CI = 0.49–0.79) (Figure 5A). Progressive disease or relapse occurred in four patients within 14 months after primary diagnosis, with no relapse afterwards. Three factors had an adverse effect on EFS in univariate analysis: female sex, TNM metastasis (M) and higher disease stage (Table 3 and Figure 5B–D). The estimated probability of OS at 5 years was 0.76 (95%CI = 0.62–0.90). Deaths occurred in three patients. Mean survival was 7.9 (95%CI = 5.8–10.0) years. Three factors had an adverse effect on OS in univariate analysis: female sex, TNM metastasis (M) and higher disease stage (Table 3).

### 3.7. Follow-Up and Causes of Deaths

Three patients experienced relapse (RFS = 80%). Two of them, with type II PPB and primary tumor R1 resection, relapsed 1 month after completing treatment and 1 year after diagnosis, respectively. Both patients died 4 and 14 months after diagnosis, respectively. The third patient, with type III PPB and a primary biopsy only, relapsed 7 months after treatment. The child received second-line chemotherapy (irinotecan + temozolomide, carboplatin + etoposide, vinblastine and trametinib) and underwent surgery with focal radiotherapy to the CNS. Three children died (OS = 80%). Two children had disease progression, both with type III PPB. The time to progression ranged from 3 to 5.5 months. One patient died due to metastases in the central nervous system (CNS) 6 months from diagnosis. The other one is on treatment, receiving second-line chemotherapy (vincristine, topotecan and cyclophosphamide).

## 4. Discussion

This study presents the clinical manifestations and treatment outcomes of PPB in the Polish pediatric population. PPB is a rare tumor, with 15 cases diagnosed in Poland between 2011 and 2024, all confirmed by at least two pathologists. After the first cases were described, the International PPB Registry was established in the United States in 1988 for central pathological verification and evaluation of clinical data and treatment outcomes. PPB is usually diagnosed in infants and young children up to 7 years of age. In our study, 93% of patients were diagnosed before the age of 4. The tumor is highly invasive and may follow an aggressive clinical course, often leading to distant metastases, disease progression or relapse after treatment. In the present study, at the time of diagnosis, respiratory failure occurred in two patients (20%), while distant metastases were identified in three patients (20%). In the International PPB/DICER1 Registry Report published in 2023, distant metastatic disease was present at diagnosis in 6% of patients, with the most common sites in bones and the CNS [17].

Due to its low incidence and clinical symptoms mimicking lower respiratory tract infections, the neoplasm may be overlooked in the differential diagnosis of pulmonary infiltrates. Type I PPB can pose diagnostic challenges and may be misdiagnosed with other lung diseases with a cystic component, such as congenital pulmonary airway malformation (CPAM), pulmonary sequestration and simple lung cysts. In children with primary lung lesions detected after birth, PPB accounts for approximately 10% of cases, with type I being the most common. Fewer than 5% of lesions are detected prenatally. Computed tomography (CT) may not always reliably differentiate congenital lung malformations from PPB; therefore, timely surgical intervention is crucial for early diagnosis and complete tumor resection.

In 2009, a pathogenic germline mutation of DICER1 was identified as a genetic background for the development of PPB. Currently, it is estimated that at least 70% of patients with PPB carry DICER1 germline pathogenic variants, which can manifest in various ways. In the study, a DICER1 mutation was confirmed in eight patients (53%), while in six patients, genetic testing was not performed. This mutation predisposes individuals to a range of malignancies and benign conditions affecting multiple tissues, including the thyroid, kidneys, liver, ovaries, eyes and brain. Genetic testing in postnatally diagnosed lung lesions may be helpful in identifying suspected cases.

In 2016, during the first meeting of the International DICER1 Symposium, a consensus was established, providing guidelines for genetic testing of patients and their families based on major and minor criteria, as well as recommendations for imaging studies of various organs for oncological surveillance. The primary goal of these recommendations is to detect PPB at the earliest cystic stage (type I), which is associated with the most favorable treatment outcomes. When a DICER1 pathogenic variant is confirmed in one parent, a fetal ultrasound is recommended during the third trimester to detect a large lung cyst that may require early surgical intervention. This imaging modality has greater sensitivity for detecting cystic changes compared to postnatal X-ray imaging. Detection of the lesion requires a chest CT even if no cyst is visible on the X-ray examination. If a cystic lesion is detected, it is recommended to remove it as soon as possible (ideally before the age of 7), taking into account the patient’s age, clinical symptoms and evolution of the lesion in subsequent examinations. Surgical intervention is a critical factor influencing treatment outcomes.

Intraoperative precautions to prevent tumor rupture and ensure complete resection with negative margins (R0) are critical. In our study group, radical primary surgery (R0) was successfully performed only in three patients (20%). It may indicate a late diagnosis and advanced PPB in most patients [22,23,24]. Within the first 2 years of disease, children are at high risk of early progression, local recurrences or fulminant CNS metastases (CWS Guidance 2014). The CNS is the most frequent site of extrathoracic metastases [25]. Patients with all initial IRS groups (from I to IV) were reported. The data suggest that a longer delay (>1 year) from diagnosis to relapse may be a favorable prognostic factor. The local treatment of CNS metastases is crucial and includes resection and focal radiotherapy. There are no guidelines for second-line treatment. Carboplatin/etoposide- and irinotecan/temozolomide-based combinations were reported as promising regimens.

In the European analysis of 112 patients diagnosed between 2000 and 2018, disease progression or recurrence occurred in 31% of cases [25]. In our cohort, it was observed in 33%. The treatment outcomes were poor, with a 5-year EFS and OS rate of 37% for the entire group [25]. In our cohort, disease progression occurred in two (13%) within 6 months from diagnosis and recurrence involving the CNS in three (20%) children within 7 months after the end of treatment. Four patients had CNS involvement, and one had multifocal lung metastases. Two patients with CNS metastatic disease had type III PPB, and two patients had type II. One patient with R0 resection in delayed surgery, type III PPB and relapsed disease in the CNS had R1 metastasis resection, radiotherapy and second-line chemotherapy with irinotecan, temozolomide, carboplatin, etoposide and, finally, vinblastine and trametinib. She is still alive. The small cohort of patients may not currently permit major, definitive conclusions. Early relapse is a well-established and adverse prognostic factor. There is a need for revised CNS imaging surveillance protocols in high-risk subgroups (e.g., type III, R1/R, DICER+). Precise monitoring using MRI every 3–6 months for 5 years enables early detection of CNS metastases. Due to the very poor prognosis of progressive or relapsed PPB disease, molecular profiling is needed to detect possible molecular-targeted treatment options [25].

All patients with type II and III PPB required a multimodal oncological treatment approach: surgery, chemotherapy and, in some cases, radiotherapy. Chemotherapy has been shown to improve treatment outcomes in type I PPB and should be considered in patients with incomplete cyst resection or intraoperative tumor rupture.

The patients received chemotherapy typically used for the treatment of high-grade soft tissue sarcomas, including vincristine, dactinomycin, cyclophosphamide, ifosfamide and doxorubicin. None of the patients underwent radiotherapy as part of first-line treatment. Radiotherapy was administered in two patients at relapse in the CNS. Oral maintenance chemotherapy (OTI-OTE) was ordered in two patients (Table 2).

In 2021, the European Cooperative Study Group for Pediatric Rare Tumors (EXPeRT), in collaboration with the European Union-funded project PARTNER (Pediatric Rare Tumors Network—European Registry), published recommendations for the diagnosis and treatment of children with PPB. Recommendations were provided regarding surgical management, chemotherapy regimens and indications for radiotherapy. In patients with R1 or R2 first surgery resection, the addition of doxorubicin was recommended. For patients who do not respond first-line treatment, new therapeutic options are actively being explored [26,27,28]. In the International PPB/DICER1 Registry Report published in 2023, data on treatment with the IVADo regimen (ifosfamid, vincristine, actinomycin, doxorubicin) and historical regimen (e.g., VAC: vincristine, actinomycin, cyclophosphamide) were compared. Non-anthracycline-containing regimens had inferior outcomes [17].

Despite the small sample size, analyzing outcomes from different chemotherapy protocols (e.g., CWS 2006, CWS 2014, EXPERT) could offer preliminary data on regimen effectiveness, acknowledging the challenges of a heterogeneous group. Additionally, the outcomes of the two patients on oral maintenance chemotherapy (OTI-OTE) need further discussion, specifically regarding its rationale, prior evidence and impact on relapse delay or reduction, as its benefit was unclear.

Metastases and a higher final stage of the disease were adverse prognostic factors for outcomes. The small sample size likely limited statistical power, making it difficult to detect additional significant associations. The heterogeneity of the study group may also have influenced the results. Patients differed in terms of histological type, disease stage, treatment strategy, and the presence of DICER1 mutations. Such variability introduces potential confounding factors that may obscure meaningful patterns. Additionally, the absence of a standardized treatment protocol further complicates the identification of prognostic factors. Differences in chemotherapy regimen and the extent of surgical resection among patients may have contributed to variability in outcomes. A more uniform therapeutic might facilitate more reliable identification of prognostic factors. These considerations highlight the challenges inherent in studying rare conditions and underscore the importance of larger, more homogeneous cohorts in future research.

Our study has clear limitations due to its retrospective nature and relatively small sample size. The presented analysis was based on clinical data reported retrospectively from eight oncology centers. Not all patients had their parameters documented and available, which could have influenced the analysis results. Some patients were transferred from other hospitals, and documentation regarding disease onset was incomplete.

In the study group, there is no visible relationship between the histological type and clinical symptoms; e.g., pneumothorax occurred in both type II (solid-cystic) and type III (solid). Respiratory failure developed both in type II and III PPB. Detection of DICER1 pathogenic variants supported the histological diagnosis of PPB and accelerated diagnosis. The small number of patients precludes confirmation of any relationship between histological type, the presence of mutations and treatment results. Two patients with disease progression had histological type III and a confirmed mutation, but recurrence occurred in both histological types with the presence or absence of the DICER1 mutation. The size of the tumor is a prognostic factor and indicates the local advancement of the disease. In the group of seven patients with tumors larger than 10 cm, all achieved a complete response (CR) after the treatment. Of the six patients initially scheduled for biopsy, only five had a tumor size > 10 cm; five had type III, and one had type II. All of them achieved R0 status after postponed surgery and CR status after completion of treatment. One patient is undergoing second-line chemotherapy due to recurrence. Based on obtained results, it can be concluded that a larger size makes the primary radical R0 procedure more difficult but does not determine the treatment effects. Many publications emphasize that the most important prognostic factors include achieving complete tumor resection (R0) and the absence of disease dissemination at the time of diagnosis. Tumor sizes > 10 cm were not significantly associated with survival outcomes. Children who underwent biopsy only, without subsequent tumor resection, had poor outcomes [17,18,26,27]. The small sample size precludes confirmation of the relationship between R2 resection and treatment results. Given the poor prognosis with incomplete resections, adjuvant radiotherapy for R1/R2 types II/III should be explored further, especially as it was only used at relapse. Large tumor sizes (>10 cm) warrant more study regarding their impact on R0 resection success and survival and their association with delayed surgery. Complete surgical resection (R0) is crucial—analyzing EFS and OS rates, comparing primary R0 to non-radical resections, as well as delayed R0 after neoadjuvant chemotherapy to primary R0, and highlighting the importance of complete resection regardless of timing.

DICER1 gene status has no effect on prognosis, but its detection accelerates and facilitates accurate diagnosis of the disease. Detection of other adverse molecular profiles, e.g., *TP53*, may assist in identifying a subset of patients at higher risk of an aggressive disease course and unfavorable prognosis. Suggestions for future research include collaborating internationally to pool data, standardizing DICER1 testing and CNS-directed therapies and exploring targeted therapies (e.g., trametinib in relapsed cases).

In conclusion, PPB may follow an aggressive clinical course and is associated with unfavorable prognosis. The possibility of PPB should be considered in young children presenting with pneumothorax, pulmonary cystic lesions or other manifestations associated with DICER1 syndrome. Pulmonary lesions (cystic, cystic/solid) should be identified early to minimize the risk of progression of PPB to more aggressive PPB subtypes.

Pediatricians should be aware of the possibility of this rare but aggressive malignant tumor in young children to achieve early detection and timely intervention. Early diagnosis and complete resection are essential to achieve a good outcome. When a DICER1 mutation is confirmed, appropriate procedures for early detection of malignant and non-malignant DICER1-associated conditions should be initiated. Clinical observation and oncological screening of the closest family is recommended [29,30,31].

## 5. Conclusions

This retrospective study describes the multimodal treatment of PPB. Early and accurate diagnosis is crucial for a good outcome. Complete surgical resection is a key prognostic factor. Genetic investigation for DICER1 pathogenic variants is useful to detect this malignant tumor in the early stages.

## Figures and Tables

**Table 1:** (**A**) Characteristics of 15 patients with PPB. (**B**) Treatment characteristic of 15 patients with PPB.

**A**													
**ID**	**Sex**	**Age at** **Diagnosis (Months)**	**Tumor Size (cm)**	**PPB Type**	**DICER1** **Gene Variant** **Nucleotide** **Sequence**	**DICER Germline/** **Somatic** **Variant**	**Initial IRS Group**	**Metastases**	**Lymph Node** **Involvement** **Due to IASLC**	**Chemotherapy**	**RT**	**PD**	**RD**
**1**	F	32	5–10	NA	NA	NA	III	M0	N0	CWS 2006 I2VAx6	CR	No	No
**2**	F	62	5–10	II	NA	NA	III	M0	N0	CWS 2014 SoTiSaR VAIA III	CR	No	Yes (CNS)
**3**	M	42	>10	III	NA	NA	IIIA	M0	N0	CWS 2006, VAIA III	CR	No	No
**4**	M	28	5–10	II	NA	NA	III	M0	N0	CWS 2006 VAIA III	CR	No	No
**5**	M	27	>10	III	NA	NA	IIIA	M0	N0	CWS 2006 VAIA III	CR	No	No
**6**	M	32	3–5	II	YES, result unavailable	Result unavailable	IIC	M0	N1 inferior mediastinum	CWS 2006 VAIA III	CR	No	No
**7**	M	36	5–10	III	NA	NA	I	M0	N1 subsegmental	CWS 2014 SoTiSaR VAIA III	CR	No	No
**8**	M	36	>10	II	Exon25 c.542G>A	Somatic	III	M0	N0	CWS 2014 SoTISaR VAIA III	CR	No	No
**9**	F	29	>10	II	NM_177438.3:c.1630C>T NM_177438.3:c.5438A>G	Somatic Somatic	IV (VHRG)	M1 paraspinally lymph nodes	N1 paraaortic, subsegmental, inferior mediastinum	CWS 2014 SoTiSaR CEVAIE	CR	No	Yes (CNS)
**10**	F	48	>10	III	No	No	III	M0	N0	CWS 2014 SoTiSaR VAIA III	CR	No	Yes (CNS)
**11**	F	40	5–10	III	Exon25 c.5438A>G p.Glu1813Gly	Somatic	IV	M1	N0	CWS 2014 SoTiSaR VAIA III	NR	Yes (CNS)	No
**12**	M	39	Unavailable, pneumo-thorax	II	NM_177438.2: c.906dup	Germline	I	M0	N0	CWS 2014 SoTiSaR IVA ×10	CR	No	No
**13**	F	39	>10	III	NM_177438.3:c.3269+1G>A NM_177438.3:c.5439G>C	Germline Somatic	III	M0	N0	CWS 2006 I2VAd ×10	CR	No	No
**14**	F	29	<5	III	YES, result unavailable	Germline	IV	M1 bone, bone marrow	N0	EXPERT IVADo x4, IVAx2	NR	Yes (lungs)	No
**15**	F	41	>10	III	Exon25 c.5439G>T p.Glu1813Asp NM_177438.3:c.[1966C>T];[=] NP_8003187.1:p.[(Arg656Ter)];[=]	Somatic Germline	IA	M0	N0	EXPERT IVA x 9	CR	No	No
**B**													
**ID**	**Primary Surgery**	**Delayed,** **Second** **Surgery**	**Results and Time of Second Surgery**			**Response** **to Treatment** **(RT)**		**Progressive** **Disease** **(PD)**		**Relapsed** **Disease** **(RD)**	**Survival Time Alive/Death Years/Months from Diagnosis**		
**1**	R2	No	-			CR		No		No	8 y, alive		
**2**	R1	No	-			CR		No		Yes (CNS)	13.5 m, death		
**3**	Biopsy	Yes	R0, after five cycles, left-sided pneumonectomia via thoracotomy			CR		No		No	10 y, alive		
**4**	R1	No	-			CR		No		No	9 y, alive		
**5**	Biopsy	Yes	R0, after five cycles, lobectomia via thoracotomy			CR		No		No	5 y, alive		
**6**	R1	No	-			CR		No		No	10 y, alive		
**7**	R0	No	-			CR		No		No	6.5 y, alive		
**8**	Biopsy	Yes	R0, after five cycles, lobectomia via thoracotomy			CR		No		No	4 y, alive		
**9**	R1 tumor rupture	No	-			CR		No		Yes (CNS)	6 m, death		
**10**	Biopsy	Yes	R0, after five cycles, lobectomia via thoracoscopy			CR		No		Yes (CNS)	5 y, alive		
**11**	Biopsy	Yes	After three cycles, lobectomia via thoracotomy			NR		Yes (CNS)		No	15 m, death		
**12**	R0	No	-			CR		No		No	5 y, alive		
**13**	Biopsy	Yes	R0, after five cycles, lobectomia via thoracoscopy			CR		No		No	9 m, alive		
**14**	R2	Yes	Lobectomia via thoracotomy			NR		Yes (lungs)		No	7 m, alive		
**15**	R0	No	-			CR		No		No	15 m, alive		

Legend. F—female; M—male; NA—no data available; CNS—central nervous system; IRS—Intergroup Rhabdomyosarcoma Study, postsurgical clinical grouping classification; PPB—pleuropulmonary blastoma, VHRG—vey-high-risk group. Quality of resection margins (based on a variant of the American Joint Committee on Cancer classification): R0—complete resection with negative margins, no malignant cells present; R1—microscopic residual disease at the surgical margin (<1 mm from tumor), incomplete microscopic resection; R2—macroscopic residual disease, incomplete resection or gross tumor left behind. Response definitions: RT—response to treatment, PD (progressive disease)—tumor growth despite therapy; RD (relapsed disease)—recurrence in locoregional, metastatic or combined sites after remission; CR (complete response)—no residual tumor visible on imaging; PR (partial response)—≥33% reduction in tumor volume; NR (no response)—<33% reduction, stable disease or progression. Clinical staging (adapted from the IRS Group system): Group I—complete resection with negative margins: IA: confined to the organ of origin; IB: with contiguous extension. Group II—gross resection with microscopic residual disease: IIA: no nodal or metastatic involvement; IIB: with resected involved lymph nodes; IIC: with incompletely resected lymph nodes and/or distant regional nodes. Group III—macroscopic residual disease: IIIA: biopsy only; IIIB: incomplete resection (>50% of tumor remaining). Group IV—distant metastases at diagnosis; includes malignant pleural effusion or pleural implants. CWS—Cooperative Weichteilsarkom Studiengruppe, CWS der GPOH, in cooperation with the European Pediatric Soft Tissue Sarcoma Study Group (EpSSG); CWS 2006—guidance for risk-adapted treatment of soft tissue sarcoma in children, adolescents and young adults, version 1.3, from 15.12.2006; CWS 2014—guidance for risk-adapted treatment of soft tissue sarcoma and soft tissue tumors in children, adolescents and young adults, version 1.6.1, from 24.05.2014; CWS Register SoTiSaR—a registry for soft tissue sarcoma and other soft tissue tumors in children, adolescents and young adults. EXPeRT—European Cooperative Study Group for Pediatric Rare Tumors; chemotherapy VAIA III: four cycles of I2Vad and five cycles of I2VA; CEVAIE—two cycles of I3VA, three cycles CEV and three cycles of I3VE; V—vincristine; I—ifosfamide; A—actinomycin; E—epirubicin; C—carboplatin; Ad—adriamycin; D—doxorubicin; oral chemotherapy OTI-OTE: T—trofosfamide, I—idarubicin, E—etoposide [12,13]; IASLC—International Association for the Study of Lung Cancer for assessment of the clinical extent of regional lymph node metastasis.

**Table 2:** Treatment overview of the study group (n = 15).

Characteristics	Patients (n = 15)
Primary surgery	Tumor resection (n = 9), biopsy only (n = 6)
Status after primary surgery	R0 (n = 3), R1 (n = 4), R2 (n = 2)
Chemotherapy	Salvage chemotherapy before diagnosis in one patient Neoadjuvant chemotherapy after biopsy, n = 6 Postoperative chemotherapy in all patients, n = 15 Oral maintenance chemotherapy O-TI/O-TE, n = 2
Radiotherapy	2 (second-line treatment)
Secondary surgery	n = 6 (R0 in n = 5; resection status not defined in one patient)

Legend: treatment recommendation according to CWS Guidance (i.e., Cooperative Soft Tissue Sarcoma Study Group) in cooperation with the International Pleuropulmonary Blastoma Registry. O-TI—oral maintenance chemotherapy, trofosfamide and idarubicin; O-TE—oral maintenance chemotherapy, trofosfamide and etoposide.

**Table 3:** Univariate analysis of risk factors for pEFS.

Factor	Characteristics	Event-Free Survival			Overall Survival		
		Events	5 y pEFS ^#^ (95%CI)	*p*-Value	Deaths	5 y OS (95%CI)	*p*-Value
Sex	Male	0/7	1.00	0.006	0/7	1.00	0.030
	Female	5/8	0.23 (0.08–0.37)		3/8	0.44 (0.24–0.63)	
Side	Left	1/6	0.83 (0.69–0.97)	0.301	0/6	1.00	0.168
	Right	4/9	0.51 (0.37–0.66)		3/9	0.64 (0.45–0.83)	
Size	<5 cm	1/2	0.50 (0.31–0.69)	0.789	0/2	1.00	0.661
	5–10 cm	2/5	0.60 (0.39–0.82)		2/5	0.60 (0.39–0.81)	
	>10 cm	2/7	0.63 (0.44–0.86)		1/7	0.89 (0.69–0.99)	
TNM tumor	1	0/3	1.00	0.161	0/3	1.00	0.280
	2	5/11	0.53 (0.36–0.70)		3/11	0.68 (0.49–0.87)	
TNM lymph nodes	0	4/12	0.65 (0.51–0.79)	0.780	2/12	0.78 (0.62–0.90)	0.541
	1	1/3	0.67 (0.42–0.91)		1/3	0.67 (0.49–0.88)	
TNM metastases	0	2/12	0.82 (0.69–0.95)	0.001	1/12	0.90 (0.69–0.99)	0.005
	1	3/3	0.00		2/3	0.00	
First surgery (R)	0	1/3	0.67 (0.42–0.91)	0.841	1/3	0.67 (0.48–0.87)	0.716
	1	1/4	0.75 (0.50–0.99)		1/4	0.67 (0.48–0.88)	
	2	3/8	0.60 (0.39–0.82)		1/8	0.83 (0.61–0.99)	
IRS grouping (version A)	I	0/2	1.00	0.013	0/2	1.00	0.047
	II	0/1	1.00		0/1	1.00	
	III	2/9	0.75 (0.58–0.84)		1/9	0.86 (0.69–0.97)	
	IV	3/3	0.00		2/3	0.00	
IRS grouping * (version B)	I–II	0/3	1.00	0.189	0/3	1.00	0.305
	III–IV	5/12	0.54 (0.36–0.69)		3/12	0.69 (0.49–0.88)	
PPB type	2	2/7	0.71 (0.49–0.92)	0.489	2/7	0.71 (0.50–0.92)	0.572
	3	3/8	0.60 (0.39–0.82)		1/8	0.80 (0.59–0.98)	
Second surgery	No	3/9	0.65 (0.49–0.82)	0.843	2/9	0.74 (0.52–0.94)	0.680
	Yes	2/6	0.63 (0.43–0.86)		1/6	0.80 (0.61–0.97)	

* in version B, groups I/II and III/IV were merged; ^#^ 5-year pEFS estimates are provided.

## Data Availability

The data presented in the study are available on request from the corresponding author. The data are not publicly available due to privacy restrictions.

## References

[1] Yu D.C., Grabowski M.J., Kozakewich H.P., Perez-Atayde A.R., Voss S.D., Shamberger R.C., Weldon C.B. (2010). Primary lung tumors in children and adolescents: A 90-year experience. J. Pediatr. Surg..

[2] Nasr A., Bass J. (2012). Thoracoscopic vs open resection of congenital lung lesions: A meta-analysis. J. Pediatr. Surg..

[3] Giuseppucci C., Reusmann A., Giubergia V., Barrias C., Krüger A., Siminovich M., Botto H., Cadario M., Boglione M., Strambach J. (2016). Primary lung tumors in children: 24 years of experience at a referral center. Pediatr. Surg. Int..

[4] Youlden D.R., Foresto S.A., Aitken J.F. (2020). Primary malignant lung tumors in children: A report from the Australian Childhood Cancer Registry, 1983–2015. Pediatr. Pulmonol..

[5] Tang J., Liu W., Li L., Liang J.H., Zeng J.H. (2022). Clinical analysis of primary lung tumors in 56 children. Zhonghua Zhong Liu Za Zhi.

[6] Neville H.L., Hogan A.R., Zhuge Y., Perez E.A., Cheung M.C., Koniaris L.G., Thompson W.R., Sola J.E. (2009). Incidence and outcomes of malignant pediatric lung neoplasms. J. Surg. Res..

[7] Messinger Y.H., Stewart D.R., Priest J.R., Williams G.M., Harris A.K., Schultz K.A., Yang J., Doros L., Rosenberg P.S., Hill D.A. (2015). Pleuropulmonary blastoma: A report on 350 central pathology-confirmed pleuropulmonary blastoma cases by the International Pleuropulmonary Blastoma Registry. Cancer.

[8] Grigoletto V., Tagarelli A., Atzeni C., Cecchetto G., Indolfi P., De Pasquale M.D., De Leonardis F., Coppadoro B., Sorbara S., Chiaravalli S. (2020). Pleuropulmonary blastoma: A report from the TREP (Tumori Rari in Età Pediatrica) Project. Tumori J..

[9] Eyssartier E., Ang P., Bonnemaison E., Gibertini I., Diot P., Carpentier E., Chantepie A., Leclair M.D., Brouard J., Boutard P. (2014). Characteristics of endobronchial primitive tumors in children. Pediatr. Pulmonol..

[10] Bisogno G., Brennan B., Orbach D., Stachowicz-Stencel T., Cecchetto G., Indolfi P., Bien E., Ferrari A., Dommange-Romero F. (2014). Treatment and prognostic factors in pleuropulmonary blastoma: An EXPeRT report. Eur. J. Cancer.

[11] Potter S.L., HaDuong J., Okcu F., Wu H., Chintagumpala M., Venkatramani R. (2019). Pediatric Bronchial Carcinoid Tumors: A Case Series and Review of the Literature. J. Pediatr. Hematol. Oncol..

[12] Hashemi A., Souzani A., Souzani A., Keshavarzi S. (2012). Pleuropulmonary blastoma in children: A case report. Iran. J. Cancer Prev..

[13] Fauroux B., Aynie V., Larroquet M., Boccon-Gibod L., Ducou le Pointe H., Tamalet A., Clément A. (2005). Carcinoid and mucoepidermoid bronchial tumours in children. Eur. J. Pediatr..

[14] Deschildre A., Sardet A., Brouard J., Delaisi B., Boussard L., Boccon-Gibod L., Gosselin B., Tournier G., Leclerc F. (1996). Bronchial mucoepidermoid carcinoma: Apropos of 3 cases. Arch. Pediatr..

[15] Karray A., Boussetta A., Sassi F., Cherifi W., Haouet S., Gargah T. (2023). Type II pleuropulmonary blastoma mistaken for rhabdomyosarcoma: A case report. Int. J. Surg. Case Rep..

[16] Okamoto M., Kimura S., Hotta M., Tsuruno Y., Fukuzawa H. (2023). Infantile type I pleuropulmonary blastoma presenting with dyspnea due to compression by pneumothorax and an occupying tumor: A case report. Surg. Case Rep..

[17] Schultz K.A.P., Harris A.K., Nelson A.T., Watson D., Lucas J.T., Miniati D., Stewart D.R., Hagedorn K.N., Mize W., Kamihara J. (2023). Outcomes for children with type II and type III pleuropulmonary blastoma following chemotherapy: A report from the International PPB/DICER1 Registry. J. Clin. Oncol..

[18] Nelson A.T., Harris A.K., Watson D., Miniati D., Finch M., Kamihara J., Mitchel S.G., Wilson D.B., Gettinger K., Rangaswami A.A. (2023). Type I and IR pleuropulmonary blastoma (PPB): A report from the International PPB/DICER1 Registry. Cancer.

[19] Spinelli C., Ghionzoli M., Sahli L.I., Guglielmo C., Frascella S., Romano S., Ferrari C., Gennari F., Conzo G., Morganti R. (2023). DICER1 syndrome: A multicenter surgical experience and systematic review. Cancers.

[20] Gonzáles I.A., Stewart D.R., Schultz K.A.P., Field A.P., Hill D.A., Dehner L.P. (2022). DICER1 tumor predisposition syndrome: An evolving story initiated with the pleuropulmonary blastoma. Mod. Pathol..

[21] Schultz K.A.P., Nelson A., Harris A.K., Finch M., Field A., Jarzembowski J.A., Wilhelm M., Mize W., Kreiger P., Conard K. (2020). Pleuropulmonary blastoma-like peritoneal sarcoma: A newly described malignancy associated with biallelic DICER1 pathogenic variation. Mod. Pathol..

[22] Schultz K.A.P., Williams G.M., Kamihara J., Stewart D.R., Harris A.K., Bauer A.J., Turner J., Shah R., Schneider K., Schneider K.W. (2018). DICER1 and associated conditions: Identification of at-risk individuals and recommended surveillance strategies. Clin. Cancer Res..

[23] Kunisaki S.M., Lal D.R., Saito J.M., Fallat M.E., St. Peter S.D., Fox Z.D., Heider A., Chan S.S., Boyd K.P., Burns R.C. (2021). Pleuropulmonary blastoma in pediatric lung lesions. Pediatrics.

[24] Engwall-Gill A.J., Chan S.S., Boyd K.P., Saito J.M., Fallat M.E., St. Peter S.D., Bolger-Theut S., Crotty E.J., Green J.R., Hulett Bowling R.L. (2022). Accuracy of chest computed tomography in distinguishing cystic pleuropulmonary blastoma from benign congenital lung malformations in children. JAMA Netw. Open.

[25] Sparber-Sauer M., Tagarelli A., Seitz G., Sorg B., Bien E., Bel-Ami T., Pourtsidis A., Almaraz R.L., Koscielniak E., Ferrari A. (2021). Children with progressive and relapsed pleuropulmonary blastoma: A European collaborative analysis. Pediatr. Blood Cancer.

[26] Bisogno G., Sarnacki S., Stachowicz-Stencel T., Colin V.M., Ferrari A., Godzinski J., Villaris M.G., Bien E., Hameury F., Helfre S. (2021). Pleuropulmonary blastoma in children and adolescents: The EXPeRT/PARTNER diagnostic and therapeutic recommendations. Pediatr. Blood Cancer.

[27] Koscielniak E., Blank B., Vokuhl C., Kazanowska B., Ladenstein R., Niggli F., Ljungman G., Handgretinger R., Seitz G., Fuchs J. (2022). Long-Term Clinical Outcome and Prognostic Factors of Children and Adolescents with Localized Rhabdomyosarcoma Treated on the CWS-2002P protocol. Cancers.

[28] Zhang N., Zeng Q., Ma X., Chen C., Yu J., Zhang X., Yan D., Xu C., Liu D., Zhang Q. (2020). Diagnosis and treatment of pleropulmonary blastoma in children: A single-center report of 41 cases. J. Pediatr. Surg..

[29] Schultz A.K., Yang J., Doros L., Williams G.M., Harris A., Stewart D.R., Messinger Y., Field A., Dehner L.P., Ashley Hill D. (2014). DICER1-Pleuropulmonary Blastoma Familiar Tumor Predisposition Syndrome: A Unique Constellation of Neoplastic Conditions. Pathol. Case Rev..

[30] Jansen F.A., Bakhuizen J., Kester L., de Krijger R.R. (2025). DICER1 Syndrome: What Do We Know of the Pathogenic Mechanism?. Cancers.

[31] Nelson A.T., Vasta L.M., Watson D., Kim J., Harris A.K., Best A.F., Harney L.A., Carr A.G., Frederickson N., Dehner L.P. (2024). Prevalence of Lung Cysts in Adolescents and Adults with a Germline DICER1 Pathogenic/Likely Pathogenic Variant: A Report from the National Institutes of Health and International Pleuropulmonary Blastoma/DICER1 Registry. Thorax.

